# Efficacy and safety comparison of small molecule anti-angiogenic drugs in the treatment of bone and soft tissue sarcomas : a network meta-analysis

**DOI:** 10.1186/s12885-025-15412-1

**Published:** 2025-12-10

**Authors:** Jianping Zhang, Yadi Liu, Xincai Zhao, Rong Xu, Cheng Guo

**Affiliations:** Department of Pharmacy, Shanghai Sixth People’s Hospital Affiliated to Shanghai Jiao Tong University School of Medicine, Shanghai, 200233 China

**Keywords:** Bone and soft tissue sarcomas, Anti-angiogenic therapy, Small molecule inhibitors, Network meta-analysis, Efficacy, Safety

## Abstract

**Background:**

Anti-angiogenic therapy, particularly small-molecule inhibitors targeting the vascular endothelial growth factor receptor (VEGFR), has emerged as a promising approach for treating bone and soft tissue sarcomas. This study aimed to systematically compare the efficacy and safety of different small-molecule anti-angiogenic agents in the treatment of bone and soft tissue sarcomas through a network meta-analysis (NMA).

**Methods:**

We conducted a comprehensive search of seven major databases, including China National Knowledge Infrastructure (CNKI), Wanfang, VIP Database (VIP), PubMed, Embase, Cochrane Library, and Web of Science, to collect clinical randomized controlled trials (RCTs) evaluating the use of small-molecule anti-angiogenic drugs in the treatment of bone and soft tissue tumors. R softwarewas used for data analysis. We combined all direct and indirect evidence to compare different treatments in terms of efficacy and safety, reported as hazard ratio (HR) for survival outcomes (progression-free survival and overall survival) and odds ratio (OR) for binary outcomes (objective response rate and disease control rate), with 95% confidence intervals (CIs). The P score was used to rank the side effect risk. This project has been registered on PROSPERO CRD42024584746.

**Results:**

The initial search yielded 946 records, and 16 studies, involving 1,291 patients, met all criteria. In terms of the disease control rate (DCR), apatinib + chemotherapy is better than chemotherapy alone (OR 0.13 (0.02, 0.92)). In terms of objective response rate (ORR), apatinib + chemo is better than pazopanib + chemotherapy (OR 0.15 (0.02, 0.94)), anlotinib is better than chemo (OR 0.26 (0.07, 0.92)) and pazopanib + chemo (OR 0.15 (0.03, 0.85)). The side effects of different treatments vary.

**Conclusions:**

Short-term efficacy of small-molecule anti-angiogenic TKIs varied across bone and soft tissue sarcomas, with trends favoring apatinib plus chemotherapy and anlotinib. However, substantial heterogeneity across studies, including sarcoma subtypes and prior therapies, limits definitive conclusions regarding comparative efficacy. A multidisciplinary team is needed to better manage the side effects.

What is new?

- Short-term efficacy varied among small-molecule anti-angiogenic TKIs in bone and soft tissue sarcomas, with trends suggesting potential benefits of apatinib combined with chemotheraphy and anlotinib.
- However, high heterogeneity across studies, including differences in sarcoma subtypes, prior therapies, and study design, limits the ability to draw definitive conclusions regarding superiority.
- Safety profiles differed among agents, and subtype-specific tumor biology, particularly angiogenesis, may influence response, highlighting the need for individualized treatment and further subtype-focused studies.

## Background

Both bone cancer and soft tissue sarcoma (STS) are considered rare diseases but their rates of diagnosis have increased in recent years [1, 2]. In 2014, the incidence of STS in China was approximately 3.17 per 100,000, resulting in approximately 43,400 new cases, with an equal distribution between men and women [3]. In the United States, an estimated 3,610 new cases of bone cancer were diagnosed in 2021, with approximately 2,060 deaths attributed to the disease [4].

Bone and soft tissue tumors exhibit significant clinical heterogeneity. Low-grade tumors generally demonstrate indolent growth with localized expansion, whereas high-grade malignancies tend to be highly aggressive, invasive, and destructive, with an increased likelihood of recurrence and distant metastasis. Owing to the complex and distinctive nature of bone and soft tissue sarcomas, which are characterized by high invasiveness and metastatic potential, overall survival remains poor despite advances in treatment [5].

The primary treatment modality for early-stage STS is surgical intervention, whereas patients with unresectable tumors or distant metastases require medical therapy [6]. Currently, anthracyclines serve as the principal first-line chemotherapy agents for advanced STS, with no established standard second-line treatment protocol [7]. Traditional first-line therapy for osteosarcoma mainly encompasses chemotherapeutic agents such as methotrexate and doxorubicin [8]. Nevertheless, these conventional chemotherapy agents manifest notable adverse effects, substantially impacting patients’ quality of life. In recent years, there has been a growing emphasis on novel small molecule drugs.

Vascular endothelial growth factor receptor (VEGFR) inhibitors, which function as anti-angiogenic agents, significantly impede osteosarcoma neovascularization by obstructing tumor vascular production. It impacts the VEGF/VEGFR pathway, inhibits the activation of angiogenesis, and ultimately blocks the replication of cancer cells [9–11].

Anti-angiogenic therapy for sarcomas encompasses both monoclonal antibodies (e.g., bevacizumab) and small-molecule tyrosine kinase inhibitors (TKIs; e.g., pazopanib, regorafenib, sorafenib, lenvatinib, anlotinib, apatinib) [12, 13]. These TKIs primarily inhibit VEGFR1-3, a key driver of tumor angiogenesis, while also targeting additional kinases involved in tumor growth: for example, pazopanib blocks PDGFR, FGFR, and KIT; regorafenib additionally inhibits RET and BRAF; sorafenib acts on CRAF and PDGFR-β; lenvatinib targets FGFR and RET along with VEGFR; and anlotinib and apatinib mainly inhibit VEGFR with some activity against PDGFR and KIT [12].

In this analysis, we focused exclusively on small-molecule TKIs, because the clinical benefit of monoclonal antibodies in metastatic soft tissue sarcomas remains uncertain. Early phase I/II studies suggested potential efficacy of bevacizumab in combination with chemotherapy, but a subsequent multicenter phase II trial (NCT00643565) failed to demonstrate a significant improvement in event-free survival compared with chemotherapy alone [14]. In contrast, small-molecule TKIs have been more extensively studied across sarcoma subtypes, providing sufficient evidence to support a network meta-analysis. Notably, pazopanib has been shown to improve median progression-free survival (mPFS) from 5 months to 10 months in certain subtypes of STS [15], whereas regorafenib has exhibited significant anti-tumor activity in patients with osteosarcoma [16].

Despite the increasing adoption of small-molecule anti-angiogenic therapies, a significant gap remains in the comparative evaluation of their efficacy and safety. Most existing studies are limited to single-arm trials or cohort studies using chemotherapy or placebo as comparators, making it challenging for clinicians to determine the optimal therapeutic choice. Given these limitations, this study aims to systematically compare the efficacy and safety of different small-molecule anti-angiogenic agents in the treatment of bone and soft tissue sarcomas through a systemic review and a network meta-analysis (NMA). By integrating direct and indirect comparisons across multiple clinical trials, this analysis seeks to provide a comprehensive evidence base to guide clinical decision-making and optimize treatment strategies for these rare but aggressive malignancies.

## Methods

We conducted a comprehensive search of seven major databases, including the China National Knowledge Infrastructure (CNKI), Wanfang, VIP Database (VIP), PubMed, Embase, Cochrane Library, and Web of Science, to collect clinical randomized controlled trials (RCTs) evaluating the use of small-molecule anti-angiogenic drugs in the treatment of bone and soft tissue tumors. The following comprehensive search strategy was used, including bone neoplasms, sarcoma, apatinib, anlotinib, pazopanib, regorafenib, sorafenib, lenvatinib, “FOCUS V”, Votrient, Stivarga, Nexavar, Lenvima. The search was conducted from the inception of the databases up to July 2024. This research was conducted following the PRISMA guidelines. This project has been registered on PROSPERO CRD42024584746.

### Inclusion and exclusion criteria

We included randomized controlled trials (RCTs) that involved patients diagnosed with primary bone tumors or soft tissue tumors. The intervention of interest was small-molecule anti-angiogenic drugs, with comparisons made to conventional treatments or placebo. The primary outcomes of interest were progression-free survival (PFS) and overall survival (OS), whereas the secondary outcomes included objective response rate (ORR), disease control rate (DCR), and the incidence of adverse events (AEs). The exclusion criteria were as follows: the full article was not available; studies with missing outcome data; studies involving non-primary bone tumors; studies with bone and soft tissue sarcoma as a subgroup analysis; studies involving desmoid tumors, gastrointestinal stromal tumors; and studies which small-molecule anti-angiogenic drugs were used as the control group.

### Data extraction

Two independent reviewers evaluated and extracted data from each study. A standardized form was used to extract the following information from each eligible article: author and publication year, cancer type, patient characteristics (number, sex, age), interventions (treatment plan, intervention time, and follow-up time), primary outcome measures (progression-free survival (PFS), and overall survival (OS)), and secondary outcome measures (objective response rate (ORR), disease control rate (DCR) and adverse event (AE) incidence). Any divergences regarding data extraction between two authors will be resolved by a third author via discussion.

### Risk of bias assessment

Two independent reviewers used the Cochrane risk of bias tool to assess the quality of the literature. Any divergences regarding data extraction between two authors will be resolved by a third author via discussion.

### Data synthesis

We conducted a network meta-analysis (NMA) to comprehensively compare and rank the safety profiles of different small molecule anti-angiogenic agents in the treatment of bone and soft tissue tumors. A network plot was generated to visually present the geometry of the evidence, including all available direct treatment comparisons. Statistical analyses were performed within a frequentist framework. Pairwise relative treatment effects with corresponding 95% confidence intervals (CIs) were summarized in a league table, enabling a systematic presentation of all head-to-head comparisons. To provide a comparative ranking of the interventions, we applied a probability-based ranking approach derived from P-scores, which quantify the extent of certainty that each treatment is better or worse than the competing options.

R software was used for data analysis. We combined all direct and indirect evidence to compare different treatments in terms of efficacy and safety, reported as hazard ratios for survival outcomes (PFS and OS) and odds ratios (ORs) for binary outcomes (ORR and DCR), with 95% confidence intervals (CIs). P score is used to rank the side effect risk. The heterogeneity test uses the Q test and I² statistic. Statistical significance was set at a P value of 0.05. The heterogeneity was judged by I². If I² < 50%, it can be considered that there is homogeneity between multiple independent studies, and a fixed model will be selected; if I² ≥ 50%, the study is considered heterogeneous, and a random model will be selected.

## Results

### Study selection

**Fig. 1:**
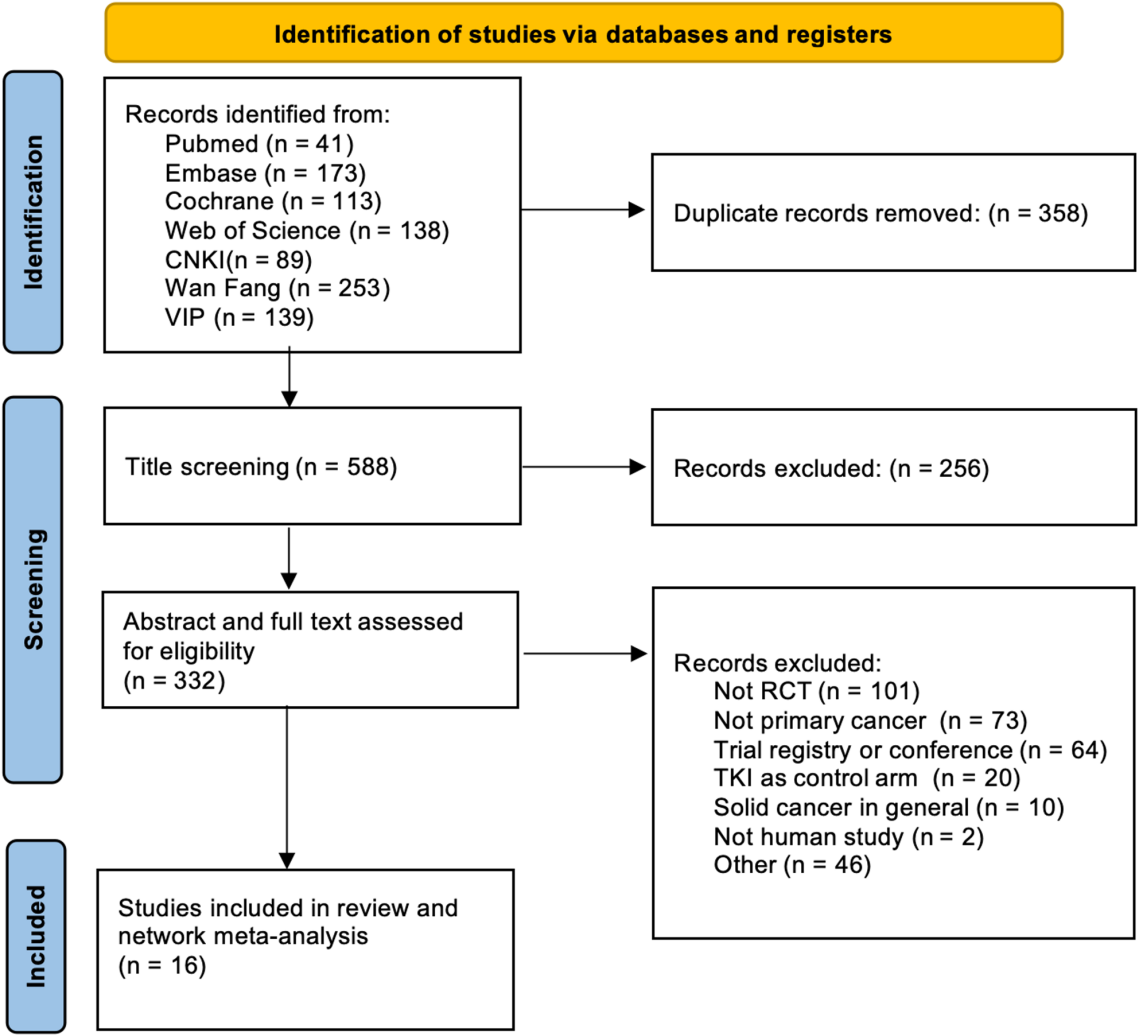
Study Selection

The initial search yielded 946 records. After 358 duplicates were removed, 332 records underwent abstract and full-text assessments for eligibility. A total of 316 articles were excluded because they did not fulfill the inclusion criteria or for other reasons. Finally, 16 studies met all the criteria and were included in the network meta-analysis. The identification and screening process is illustrated in the PRISMA diagram in Fig. 1.

### Methodological quality of the included studies

**Fig. 2:**
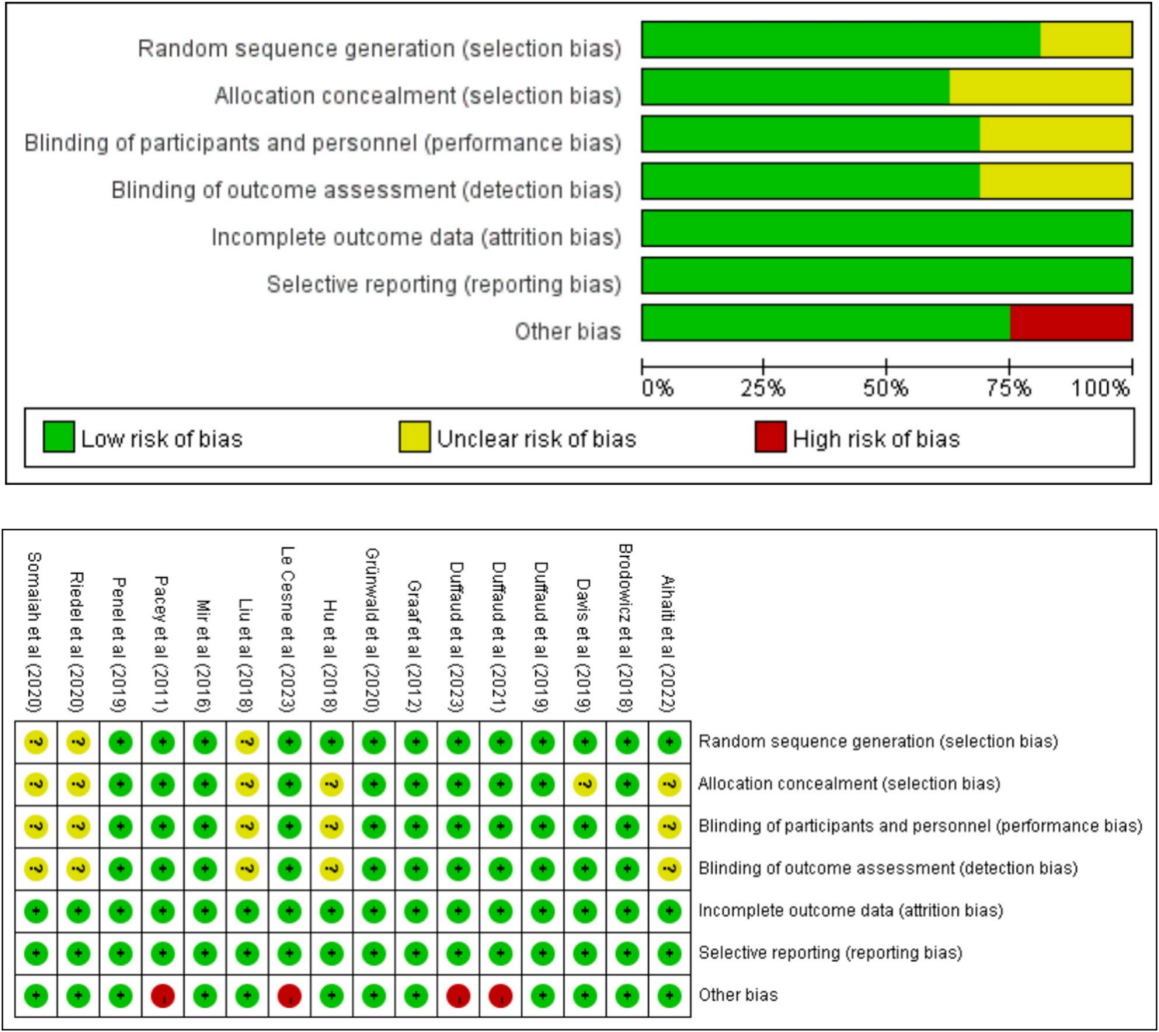
Quality of Included Studies

A total of 16 included studies were assessed via the Cochrane risk of bias tool to assess the quality of the literature. The detailed evaluation results for each item are shown in Fig. 2.

### Description of included studies

Although our network meta-analysis can provide comparative insights into TKI efficacy and safety, the heterogeneity of sarcoma subtypes and limited trial numbers constrain subtype-specific conclusions. Therefore, we also present a descriptive summary of TKI performance across different bone and soft tissue sarcomas, highlighting observed efficacy, tolerability, and prior treatment contexts.

Sixteen RCTs involving 1,291 patients were included. Four studies focused on bone tumors [15–18], one focused on Ewing Sarcoma originating in bone [19], whereas the remaining studies investigated soft tissue sarcomas [20–30]. These studies were published between 2011 and 2023. In general, male and female participants were evenly distributed, although patient age varied depending on the specific cancer type. The sample sizes ranged from a minimum of 5 to a maximum of 369 participants. In terms of treatment regimens, nine studies analyzed regorafenib (160 mg once daily, PO, 21 days on, 7 days off, 28-day cycle), two studies examined anlotinib (12 mg once daily, PO, 14 days on, 7 days off, 21-day cycle), three studies investigated pazopanib (800 mg once daily, PO, 21-day cycle), one study assessed apatinib (500 mg once daily, PO), and one study evaluated sorafenib (400 mg twice daily, PO). The characteristics of the included studies are summarized in Table 1.

**Table 1:** Characteristics of included studies

Year	Author	Cancer type	Previous Treatment	Participants					Treatment^		Reported outcomes
				Age* (I/C^#^)		Male%	*N* (I/C)		Intervention	Control	
2019	Duffaud et al.	Metastatic Osteosarcoma	Doxorubicin, Ifosfamide, Cisplatin, High-dose methotrexate, Etoposide, Gemcitabine or docetaxel, Oral cyclophosphamide	32	40	63%	26	12	Regorafenib	Placebo	ORR DCR
2019	Davis et al.	Advanced or metastatic bone or extraskeletal osteosarcoma	at least 1 prior line of systemic therapy in the neoadjuvant, adjuvant, or metastatic setting	33	47	48%	22	20	Regorafenib	Placebo	PFS OS
2021	Duffaud et al.	Metastatic or Locally Advanced Chondrosarcoma	1–2 previous lines of chemotherapy	64	53	63%	24	16	Regorafenib	Placebo	ORR DCR
2023	Le Cesne et al.	Relapsed Advanced or Metastatic Chordoma	0–2 prior systemic regimens (either chemotherapy or targeted therapy)	67.5	54	70%	16	7	Regorafenib	Placebo	DCR
2023	Duffaud et al.	Ewing Sarcoma originating in bone	1–2 previous chemotherapy regimens	32	28	78%	23	13	Regorafenib	Placebo	ORR
2011	Pacey et al.	Advanced Soft Tissue Sarcoma (Unspecified)	No limit on the extent of prior therapy	52	67	20%	3	2	Sorafenib	Placebo	ORR DCR
2012	Graaf et al.	Metastatic Soft Tissue Sarcoma (Leiomyosarcoma; Synovial sarcoma; Other)	Anthracycline and a maximum of four previous lines of systemic therapy (no more than two lines of combination regimens)	56.7	51.9	41%	246	123	Pazopanib	Placebo	PFS OS ORR DCR
2016	Mir et al.	Liposarcoma	Previous doxorubicin or other anthracycline treatment	57	65	60%	20	23	Regorafenib	Placebo	PFS ORR DCR
		Leiomyosarcoma		60	60	29%	28	28	Regorafenib	Placebo	
		Synovial Sarcoma		46	35	63%	13	14	Regorafenib	Placebo	
		Other Sarcomas (Not gastrointestinal stromal tumour, peripheral neurectodermal tumour, alveolar or embryonal rhabdomyosarcoma, primary bone)		60	55	59%	29	27	Regorafenib	Placebo	
2018	Brodowicz et al.	Advanced Non-Lipogenic Soft Tissue Sarcoma (Leiomyosarcoma; Synovial Sarcoma; Other nonadipocytic sarcoma)	Doxorubicin or other	Not mentioned	Not mentioned	47%	70	69	Regorafenib	Placebo	PFS OS ORR DCR
2018	Liu et al.	Advanced Soft Tissue Sarcoma (Synovial Sarcoma; Alveolar Soft Part Sarcoma; Leiomyosarcoma; Undifferentiated Pleomorphic Sarcoma; Liposarcoma; Other)	Anthracyclines-based therapy	39.8	44	59%	28	11	Anlotinib	Placebo	ORR DCR
2018	Hu et al.	Advanced Soft Tissue Sarcoma (Unspecified)	Unspecified	54	55	64%	21	21	Apatinib + AIM (Doxorubicin, Ifosfamide, Mesna)	AIM	ORR DCR
2019	Penel et al.	Advanced Non-Lipogenic Soft Tissue Sarcoma (Leiomyosarcoma; Synovial sarcoma; Undifferentiated pleomorphic Sarcoma; Angiosarcoma; Solitary fibrous tumour; Spindle cell osseous sarcoma)	Doxorubicin, Ifosfamide, Trabectedin, Dacarbazine, Gemcitabine, Taxane, Cyclophosphamide	61	60	24%	18	19	Regorafenib	Placebo	PFS OS ORR DCR
2020	Riedel et al.	Advanced Metastatic Refractory Liposarcoma (Dedifferentiated liposarcoma; Myxoid and/or round cell liposarcoma; Pleomorphic liposarcoma)	Unspecified	61.22	64.17	58%	24	24	Regorafenib	Placebo	PFS OS ORR
2020	Grünwald et al.	Soft Tissue Sarcoma (fibrosarcoma, pleomorphic high-grade sarcoma, leiomyosarcoma, liposarcoma, alveolar or pleomorphic rhabdomyosarcoma, vascular sarcoma, synovial sarcoma not otherwise specified, and malignant peripheral nerve sheath tumors)	Previous anthracycline-based chemotherapy with curative intent was permitted if it had been completed more than 6 months before recurrence.	72	70	51%	81	39	Pazopanib	Doxorubicin	PFS OS ORR DCR
2021	Somaiah et al.	Advanced Soft Tissue Sarcoma (Unspecified)	Prior pelvic radiotherapy; Surgery; doxorubicin-based therapy	57.93	54.6	49%	45	45	Pazopanib + Gemcitabine	Gemcitabine + Docetaxel	PFS OS ORR DCR
2022	Aihaiti et al.	Advanced Soft Tissue Sarcoma (Unspecified)	First-line chemotherapy	62.58	62.72	60%	21	21	Anlotinib	Liposomal Doxorubicin + Ifosfamide	ORR DCR

*qd* once daily, *PO* by mouth, *OS* Overall Survival, *PFS* Progression-Free Survival, *DCR* Disease Control Rate, *ORR* Objective Response Rate

* Mean age or median age, determined by the study reported data

^#^ Intervention arm: Control arm

^ TKI regimens include agent, dose, and schedule as reported in each study. Detailed dosing and administration for each TKI are provided below. Anlotinib: 12 mg qd PO, 14 days on, 7 days off, 21-day cycle; Regorafenib: 160 mg qd PO, 21 days on, 7 days off, 28-day cycle; Sorafenib: 400 mg bid PO; Pazopanib: 800 mg qd PO; Apatinib: 500 mg qd

The included studies collectively investigated the efficacy and safety of small-molecule TKIs in a broad spectrum of bone and soft tissue sarcomas, encompassing both common and rare histological subtypes. For bone sarcomas, the studies included metastatic osteosarcoma, advanced or metastatic extraskeletal osteosarcoma, locally advanced or metastatic chondrosarcoma, relapsed advanced chordoma, and Ewing sarcoma originating in bone, with patients generally having received 1–2 prior chemotherapy regimens or, in some cases, multiple prior lines including high-dose methotrexate, doxorubicin, cisplatin, ifosfamide, or gemcitabine/docetaxel. Among soft tissue sarcomas, study populations included leiomyosarcoma, liposarcoma, synovial sarcoma, undifferentiated pleomorphic sarcoma, angiosarcoma, alveolar soft part sarcoma, solitary fibrous tumor, spindle cell sarcoma, fibrosarcoma, and other high-grade non-lipogenic sarcomas. The majority of these patients had received prior systemic therapy, typically anthracycline-based regimens, with some studies permitting up to four previous lines of treatment.

Small-molecule TKIs, including anlotinib, regorafenib, pazopanib, and apatinib, demonstrated variable efficacy across sarcoma subtypes. In non-adipocytic soft tissue sarcomas, anlotinib consistently prolonged progression-free survival with manageable toxicity [21, 23], while regorafenib showed meaningful activity in heavily pretreated non-adipocytic STS [24, 26, 28, 30], and pazopanib provided an alternative to chemotherapy in selected patients [29]. Apatinib combined with chemotherapy also showed improved short-term outcomes [22] In bone sarcomas, regorafenib exhibited clinically meaningful activity in relapsed or metastatic osteosarcoma [16, 19] and modest disease control in chondrosarcoma [17] and Ewing sarcoma [20], though benefits in chordoma were limited [18]. Overall, efficacy appeared dependent on histological subtype and prior therapy, highlighting the need for subtype-specific studies to guide TKI use in sarcomas.

### Network meta-analysis

**Fig. 3:**
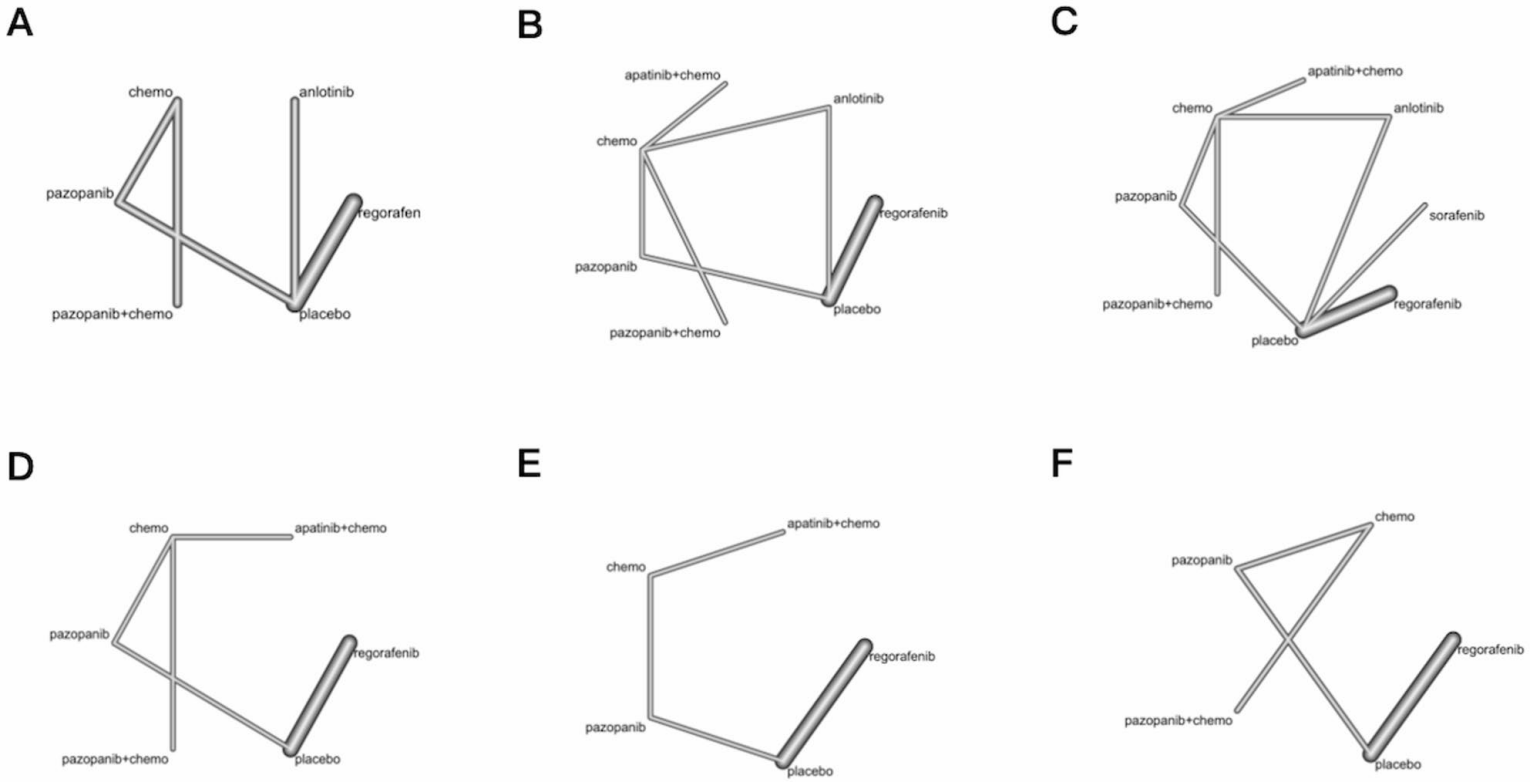
Network diagrams of comparisons on different outcomes of treatments in patients with bone or soft tissue tumors. Note: Efficacy Outcomes: (**A**) Overall Survival (OS) and Progression-Free Survival (PFS), (**B**) Disease Control Rate (DCR), (**C**) Objective Response Rate (ORR). Treatment-Related Toxicities (Grade ≥ 3): (**D**) Hypertension, (**E**) Diarrhea, (**F**) Asthenia

The network meta-analysis included five treatments (pazopanib, pazopanib + chemotherapy, chemotherapy, anlotinib, and regorafenib) for OS and PFS. Six treatments (pazopanib, pazopanib + chemotherapy, chemotherapy, anlotinib, regorafenib, and apatinib + chemotherapy) were used to determine the DCR and ORR. Five treatments (pazopanib, pazopanib + chemotherapy, chemotherapy, apatinib + chemotherapy, regorafenib) were used for hypertension, and four treatments were used for diarrhea (pazopanib, chemotherapy, apatinib + chemotherapy, regorafenib) and asthenia (pazopanib, pazopanib + chemotherapy, chemotherapy, regorafenib). The network diagrams are shown in the Fig. 3.

In terms of overall survival and progression-free survival, similar effects were observed for pazopanib, pazopanib + chemotherapy, chemotherapy, anlotinib, and regorafenib because the hazard ratios were close to 1. In terms of the DCR, apatinib + chemotherapy was better than chemotherapy alone (OR 0.13 (0.02; 0.92)). In terms of the ORR, apatinib + chemotherapyis better than pazopanib + chemotherapy (OR 0.15 (0.02; 0.94)), anlotinib is better than chemo (OR 0.26 (0.07; 0.92)) and pazopanib + chemotherapy(OR 0.15 (0.03; 0.85)). The details are shown in Table 2.

**Table 2:** Pooled estimates of the network Meta-analysis

A. Overall Survival (OS)					
regorafenib					
0.97(0.75;1.26)	chemo				
0.94(0.80;1.11)	0.97(0.79;1.18)	pazopanib			
0.94(0.59;1.48)	0.96(0.59;1.58)	0.99(0.63;1.56)	anlotinib		
0.89(0.63;1.25)	0.91(0.73;1.14)	0.95(0.70;1.27)	0.95(0.55;1.63)	pazopanib + chemo	
**0.88(0.78;1.00)**	0.91(0.72;1.14)	0.94(0.84;1.05)	0.94(0.61;1.46)	0.99(0.72;1.36)	placebo

B. Progression-Free Survival (PFS)					
chemo					
0.99 (0.72;1.37)	pazopanib				
0.98 (0.49;1.96)	0.99 (0.53;1.82)	anlotinib			
0.91 (0.65;1.28)	0.92 (0.58;1.47)	0.93 (0.43;2.02)	pazopanib + chemo		
0.81 (0.52;1.28)	0.82 (0.60;1.13)	0.83 (0.48;1.46)	0.89 (0.51;1.57)	regorafenib	
**0.60 (0.39;0.92)**	**0.60 (0.45;0.80)**	0.61 (0.35;1.05)	0.65 (0.38;1.13)	**0.73 (0.64;0.84)**	placebo

C. Disease Control Rate (DCR)						
placebo						
0.45 (0.08;2.37)	chemo					
0.45 (0.04;4.41)	1.00 (0.21;4.81)	pazopanib+chemo				
**0.27 (0.07;0.95)**	0.60 (0.15;2.35)	0.60 (0.07;4.80)	pazopanib			
**0.24 (0.13;0.45)**	0.54 (0.09;3.19)	0.54 (0.05;5.78)	0.90 (0.22;3.73)	regorafenib		
**0.19 (0.04;0.99)**	0.43 (0.08;2.22)	0.43 (0.04;4.15)	0.71 (0.12;4.14)	0.79 (0.14;4.65)	anlotinib	
**0.06 (0.00;0.75)**	**0.13 (0.02;0.92)**	0.13 (0.01;1.58)	0.21 (0.02;2.36)	0.23 (0.02;3.38)	0.29 (0.02;3.89)	apatinib+chemo

**D. Objective Response Rate (ORR)**							
placebo							
0.38 (0.03;4.76)	pazopanib+chemo						
0.41 (0.16;1.08)	1.09 (0.07;16.38)	regorafenib					
0.33 (0.01;12.82)	0.88 (0.01;74.91)	0.81 (0.02;35.14)	sorafenib				
0.23 (0.03;2.08)	0.62 (0.12;3.07)	0.56 (0.05;6.17)	0.70 (0.01;49.33)	pazopanib			
0.22 (0.02;2.03)	0.58 (0.17;1.93)	0.53 (0.05;5.99)	0.66 (0.01;47.18)	0.94 (0.32;2.71)	chemo		
**0.06 (0.00;0.78)**	**0.15 (0.02;0.94)**	0.14 (0.01;2.24)	0.17 (0.00;15.20)	0.24 (0.04;1.39)	0.26 (0.06;1.03)	apatinib+chemo	
**0.06 (0.01;0.52)**	**0.15 (0.03;0.85)**	0.14 (0.01;1.53)	0.17 (0.00;12.05)	0.24 (0.05;1.18)	**0.26 (0.07;0.92)**	0.99 (0.15;6.49)	anlotinib

Hazard ratios (HRs) with 95% CIs were used for survival outcomes (PFS and OS); odds ratios (ORs) with 95% CIs were used for binary outcomes (ORR and DCR)

Table 3 shows the p score for different side effects. With respect to the side effects, regorafenib, pazopanib, and pazopanib + chemotherapy were among the three drugs associated with the highest risk of developing hypertension. In terms of diarrhea risk, apatinib + chemotherapy > chemotherapy > pazopanib > regorafenib. As for asthenia risk, pazopanib > regorafenib > chemotherapy > pazopanib + chemotherapy. Notably, the risk of asthenia associated with pazopanib + chemo is lower than that associated with the placebo.

**Table 3:** P-score results table for treatment-related toxicities (Grade ≥ 3)

Treatment	HTN (Rank)	Diarrhea (Rank)	Asthenia (Rank)
chemo	0.9349 (1st)	0.3398 (4th)	0.5102 (3rd)
apatinib + chemo	0.6886 (2nd)	0.1659 (5th)	—
placebo	0.5367 (3rd)	0.9516 (1st)	0.6755 (2nd)
pazopanib + chemo	0.4603 (4th)	—	0.8891 (1st)
pazopanib	0.2913 (5th)	0.4345 (3rd)	0.1808 (5th)
regorafenib	0.0882 (6th)	0.6082 (2nd)	0.2443 (4th)

“—” indicates missing data for that toxicity comparison. Higher p-score means better safety

## Discussion

This study compared the efficacy and safety of different small-molecule anti-angiogenic drugs in the treatment of bone and soft tissue sarcomas through a network meta-analysis. The results revealed significant differences among these drugs in terms of short-term efficacy indicators (DCR and ORR). Apatinib combined with chemotherapy and anlotinib demonstrated superior performance in terms of DCR and ORR, which may be attributed to their distinct mechanisms and pharmacokinetic properties. The high selectivity of apatinib for VEGFR-2 may enable more effective short-term blockade of tumor angiogenesis, thereby rapidly inhibiting tumor growth and improving short-term efficacy [31]. Additionally, apatinib and anlotinib have longer half-lives and stable plasma concentrations, which may be more conducive to the continuous inhibition of angiogenesis [32, 33]. In contrast, regorafenib has a faster metabolism and requires a higher dose to achieve the same effect [34]. Moreover, the combination with chemotherapy may further enhance the anti-tumor effect through synergistic action [35]. Chemotherapy drugs may disrupt the tumor cell matrix, thereby increasing the permeability and targeting of small-molecule drugs and thus improving short-term efficacy.

Short-term efficacy indicators (DCR and ORR) hold significant clinical value in the treatment of bone and soft tissue sarcomas. These types of sarcomas progress rapidly, and patients in the advanced stages have limited treatment options [1, 2]. A higher disease control rate in the short term can reduce the tumor burden and rapidly alleviate symptoms such as pain and bleeding, thereby improving the quality of life for patients. This study suggests that apatinib in combination with chemotherapy or anlotinib may be more suitable for patients who require rapid tumor shrinkage.

However, differences in long-term efficacy (such as PFS and OS) among different small-molecule anti-angiogenic drugs in the treatment of bone and soft tissue tumors are not significant. This may be related to the tumor heterogeneity, acquired resistance, and the impact of subsequent therapies. Bone and soft tissue tumors are highly heterogeneous, with complex and diverse angiogenesis mechanisms [36]. Although small-molecule anti-angiogenic drugs can inhibit tumor angiogenesis in the short term, long-term use may lead to drug resistance in tumors through the activation of bypass signaling pathways or adaptation to the microenvironment, resulting in similar long-term efficacy [37, 38]. In addition, subsequent treatment regimens (such as second-line treatments) may also have an important impact on long-term survival, thereby masking the differences in initial treatments [7].

In addition, our study investigated the safety profile of different treatments in particular. Regorafenib and pazopanib are more likely to cause hypertension. The mechanism is directly related to the inhibition of endothelium-derived relaxing factors, capillary rarefaction and alterations in the pressure natriuresis relationship [39, 40]. Although dose modifications are permitted to help manage patient tolerance, regorafenib and pazopanib still present the highest risk for hypertension. For the outcome of asthenia, the network estimate was driven by a single randomized trial in which the “chemotherapy” node corresponded specifically to gemcitabine–docetaxel [24]. Thus, the higher risk of asthenia observed with chemotherapy versus pazopanib plus chemotherapy likely reflects the toxicity profile of this doublet rather than an interaction with pazopanib.This underscores a limitation of the analysis and necessitates cautious interpretation.

Given the distinct safety profiles of these medications, there is a clear need for better dose tapering strategies and more effective management plans for side effects [41]. Currently, there are no evidence-based guidelines for the management of anti-angiogenesis agent-induced hypertension. A clearer and more effective protocol for managing hypertension requires further study. When designing a hypertension management plan, we should consider the following details [41, 42]: (1) the target blood pressure before and during treatment, (2) medications to avoid worsening hypertension, (3) medications specifically used to treat hypertension, (4) the frequency of active monitoring and the follow-up plan, and (5) a plan for dose modification or tapering. Given the complexity of disease-drug management and the proven value of pharmacists in managing side effects [43–45], we encourage the involvement of pharmacists in this management service. Our study also revealed that regorafenib has the lowest diarrhea risk, and pazopanib + chemo has the lowest asthenia risk. Clinicians could initiate a conversation with patients before treatment to develop a patient-centered treatment plan.

Owing to the drug resistance and frequent side effects associated with anti-angiogenic therapies, there is a growing interest in studies exploring combination strategies that integrate anti-angiogenic agents with immunotherapy [46]. The advantages of combination therapy lie in the synergistic effects [47]. Moving forward, we hope to conduct further efficacy and safety analyses on combination treatment regimens.

Our review has the following strengths. First, our review thoroughly searched seven databases, including international and Chinese databases. This increases the universality and generalizability of data. Second, our study is the first to compare different bone and soft tissue sarcoma treatment plans. It provides a baseline for clinicians to choose between the treatments. Third, we emphasized the pharmacist’s role in managing therapy side effects and called for multidisciplinary cooperation to care for patients.

This review has several limitations that should be taken into account. Firstly, the rebound after the treatment cannot be measured and excluded. Because of the invasiveness of the tumor and the patient safety, patients are usually given other treatments afterward. This may diminish the efficacy of specific treatments and make it difficult to compare different treatments. Second, some of the studies had small sample sizes and short follow-up times, which may make it difficult to determine the PFS and OS benefits of anti-angiogenic agents.

Third, soft tissue and bone sarcomas are highly heterogeneous, with distinct biology and clinical behavior across subtypes. Most available clinical trials report aggregated outcomes for “STS” or “bone sarcoma” populations rather than individual subtypes, limiting the ability to draw subtype-specific conclusions regarding TKI efficacy. To enhance transparency, we summarized the histological subtypes included in each trial (Table 1) and provided a textual description to aid interpretation. Although most trials report aggregated outcomes, our network meta-analysis synthesizes the currently available evidence and provides meaningful insights into the overall comparative safety and activity of small-molecule anti-angiogenic TKIs. The variable efficacy observed across sarcoma subtypes may be explained by differences in tumor angiogenesis. Angiogenic biomarkers, particularly VEGF, play a key role in sarcoma progression. Elevated VEGF expression is associated with higher tumor grade, advanced stage, larger tumor size, and worse prognosis in both osteosarcoma and soft tissue sarcomas [48–50]. However, VEGF expression is heterogeneous across histological subtypes: it is frequently upregulated in osteosarcoma, high-grade soft tissue sarcomas, and angiosarcoma, variably expressed in liposarcoma, and less consistently detected in epithelioid sarcoma [51–54]. These findings provide a biological rationale for the differential responses to anti-angiogenic TKIs and highlight the need for subtype-specific biomarker studies to optimize patient selection.

## Conclusion

This network meta-analysis compared the efficacy and safety of different small-molecule anti-angiogenic TKIs in bone and soft tissue sarcomas. Our findings indicate that apatinib combined with chemotherapy and anlotinib may offer superior short-term efficacy (DCR and ORR), whereas long-term outcomes (PFS and OS) are generally comparable across agents, likely due to tumor heterogeneity, acquired resistance, and subsequent therapies. Safety profiles differ among drugs: regorafenib and pazopanib are associated with higher hypertension risk, while regorafenib shows lower diarrhea incidence and pazopanib plus chemotherapy has lower asthenia risk, underscoring the importance of individualized management and pharmacist involvement.

The observed variability in TKI efficacy across sarcoma subtypes may be explained by differences in tumor angiogenesis. VEGF expression, a key angiogenic biomarker, is elevated in osteosarcoma, high-grade soft tissue sarcomas, and angiosarcoma, but heterogeneous across other subtypes, providing a biological rationale for differential responses and emphasizing the need for subtype-specific biomarker studies.

Overall, our study offers a comparative overview of small-molecule anti-angiogenic TKIs in sarcomas, highlighting agents that may be preferable for rapid disease control, informing clinical decision-making, and identifying areas for future research, including biomarker-guided therapy and combination strategies with immunotherapy.

## Data Availability

Not applicable.

## Abbreviations

VEGFR: Vascular endothelial growth factor receptor
NMA: Network meta-analysis
CNKI: China National Knowledge Infrastructure
VIP: VIP Database
RCTs: Randomized controlled trials
CI: Confidence interval
STS: Soft tissue sarcoma
TKIs: Tyrosine kinase inhibitors
mPFS: Median progression-free survival
PFS: Progression-free survival
OS: Overall survival
ORR: Objective response rate
DCR: Disease control rate
AEs: Adverse events
QD: Once daily
PO: By mouth
HR: Hazard ratio
OR: Odds ratio
